# Scalable high-temperature superconducting diodes enabled by intrinsic Josephson junctions

**DOI:** 10.1093/nsr/nwag285

**Published:** 2026-05-22

**Authors:** Zihan Wei, Youkai Qiao, Yang-Yang Lyu, Da Wang, Tianyu Li, Leonardo Rodrigues Cadorim, Ping Zhang, Wen-Cheng Yue, Dingding Li, Ziyu Song, Zixi Wang, Yunfan Wang, Milorad V Milošević, Yong-Lei Wang, Huabing Wang, Peiheng Wu

**Affiliations:** Research Institute of Superconductor Electronics (RISE) & Key Laboratory of Optoelectronic Devices and Systems with Extreme Performances of MOE, School of Electronic Science and Engineering, Nanjing University, Nanjing 210023, China; Purple Mountain Laboratories, Nanjing 211111, China; National Laboratory of Solid State Microstructures & School of Physics, Nanjing University, Nanjing 210023, China; Research Institute of Superconductor Electronics (RISE) & Key Laboratory of Optoelectronic Devices and Systems with Extreme Performances of MOE, School of Electronic Science and Engineering, Nanjing University, Nanjing 210023, China; National Laboratory of Solid State Microstructures & School of Physics, Nanjing University, Nanjing 210023, China; Research Institute of Superconductor Electronics (RISE) & Key Laboratory of Optoelectronic Devices and Systems with Extreme Performances of MOE, School of Electronic Science and Engineering, Nanjing University, Nanjing 210023, China; COMMIT, Department of Physics, University of Antwerp, Antwerp 2000, Belgium; Research Institute of Superconductor Electronics (RISE) & Key Laboratory of Optoelectronic Devices and Systems with Extreme Performances of MOE, School of Electronic Science and Engineering, Nanjing University, Nanjing 210023, China; Purple Mountain Laboratories, Nanjing 211111, China; Research Institute of Superconductor Electronics (RISE) & Key Laboratory of Optoelectronic Devices and Systems with Extreme Performances of MOE, School of Electronic Science and Engineering, Nanjing University, Nanjing 210023, China; Research Institute of Superconductor Electronics (RISE) & Key Laboratory of Optoelectronic Devices and Systems with Extreme Performances of MOE, School of Electronic Science and Engineering, Nanjing University, Nanjing 210023, China; Purple Mountain Laboratories, Nanjing 211111, China; Research Institute of Superconductor Electronics (RISE) & Key Laboratory of Optoelectronic Devices and Systems with Extreme Performances of MOE, School of Electronic Science and Engineering, Nanjing University, Nanjing 210023, China; Research Institute of Superconductor Electronics (RISE) & Key Laboratory of Optoelectronic Devices and Systems with Extreme Performances of MOE, School of Electronic Science and Engineering, Nanjing University, Nanjing 210023, China; Research Institute of Superconductor Electronics (RISE) & Key Laboratory of Optoelectronic Devices and Systems with Extreme Performances of MOE, School of Electronic Science and Engineering, Nanjing University, Nanjing 210023, China; COMMIT, Department of Physics, University of Antwerp, Antwerp 2000, Belgium; Research Institute of Superconductor Electronics (RISE) & Key Laboratory of Optoelectronic Devices and Systems with Extreme Performances of MOE, School of Electronic Science and Engineering, Nanjing University, Nanjing 210023, China; Purple Mountain Laboratories, Nanjing 211111, China; State Key Laboratory of Spintronics Devices and Technologies, Nanjing University, Nanjing 210093, China; Research Institute of Superconductor Electronics (RISE) & Key Laboratory of Optoelectronic Devices and Systems with Extreme Performances of MOE, School of Electronic Science and Engineering, Nanjing University, Nanjing 210023, China; Purple Mountain Laboratories, Nanjing 211111, China; Research Institute of Superconductor Electronics (RISE) & Key Laboratory of Optoelectronic Devices and Systems with Extreme Performances of MOE, School of Electronic Science and Engineering, Nanjing University, Nanjing 210023, China; Purple Mountain Laboratories, Nanjing 211111, China

**Keywords:** high-temperature superconducting diodes, intrinsic Josephson junctions, tunable nonreciprocity, zero-field memory effect, scalability

## Abstract

Superconducting diodes, enabling nonreciprocal supercurrents, hold promise for dissipationless electronics and time-reversal-symmetry-broken physics. Developing platforms that combine high-temperature operation with scalable fabrication remains a critical challenge for the field. Here, we show that the intrinsic Josephson junctions naturally in the layered cuprate Bi_2_Sr_2_CaCu_2_O_8+δ_ offer a robust, lithography-compatible platform for high-temperature superconducting diodes. By controlling the number of naturally stacked junctions, we achieve tunable nonreciprocity, with single-surface junctions exhibiting peak efficiency and programmable zero-field memory states. A microscopic model attributes this behavior to geometry-induced anharmonicity in the current-phase relation, amplified by atomic-scale barriers. Moreover, by exploiting the natural junction architecture, we fabricate arrays containing hundreds of reproducible diodes. By uniting high-temperature operation with scalability and programmable functionality, intrinsic Josephson diodes establish a practical route toward superconducting electronics and open new avenues of nonreciprocal superconducting transport.

## Teaser

This research presents a scalable high-temperature superconducting diode based on intrinsic Josephson junctions of cuprates, with record-high operating temperatures, high rectification efficiency and programmable memory effect for next-generation energy-efficient electronics.

## INTRODUCTION

Nonreciprocal charge transport—the ability to preferentially conduct electrical current in one direction—is central to modern electronics, enabling essential components such as rectifiers, voltage regulators, and photodetectors. Extending this concept into superconductors promises dissipationless counterparts of these devices, offering a pathway for ultra-low-power information technologies [1–4]. The superconducting diode effect, wherein supercurrent exhibits directional asymmetry, has now been realized in a variety of systems, including superlattices [5–7], two-dimensional materials [8–10] and microbridges [11–13]. Among these platforms, the Josephson junction, formed by sandwiching a thin nonsuperconducting barrier between two superconductors, has emerged as a versatile unit capable of supporting diverse designs and tunable functionalities [14–25]. Implementations have ranged from planar junctions [14–16], magnetic-atom junctions [17], van der Waals (vdW) heterostructures [18–20], to superconducting quantum interference devices [21–23] and multiterminal devices [24,25].

Most reported Josephson diodes rely on low-transition-temperature (*T*_c_) superconductors, thereby constraining their operating range. Pushing the diode effects into high-*T*_c_ systems is crucial for reducing cooling demands and advancing practical integration. The cuprate superconductor Bi_2_Sr_2_CaCu_2_O_8+δ_ (BSCCO), with its strongly layered structure, offers an attractive platform for such effects [26–30]. Recent studies have employed twisted BSCCO flakes to create artificial Josephson junctions [31], revealing twist-angle-dependent diode behavior [26–29]. These works established the feasibility of high-temperature superconducting diodes. However, a persistent challenge within the field remains the development of scalable fabrication platforms that simultaneously enable high-temperature operation while maintaining manufacturability at scale.

**Figure 1.**
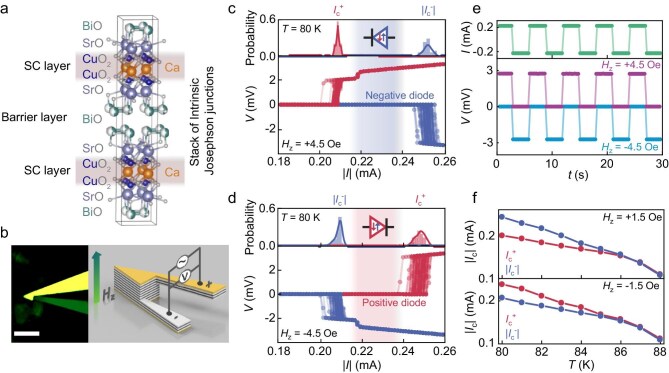
High-temperature intrinsic Josephson diode effect. (a) Crystal structure of BSCCO, composed of alternating superconducting CuO_2_ bilayers (SC layer) and insulating SrO-BiO barriers, which naturally form IJJs. (b) Optical image and schematic diagram of the intrinsic Josephson diode. Scale bar: 30 μm. (c and d) Nonreciprocal supercurrent transport at 80 K under the magnetic field of +4.5 Oe (c) and −4.5 Oe (d), respectively. The lower panels display 100 repeated current-voltage sweeps with positive and negative currents. The upper panels show statistical distributions of the superconducting critical currents *I*_c_^+^ and |*I*_c_^−^|. (e) Half-wave rectification at 80 K for *H*_z_=+4.5 Oe and *H*_z_=−4.5 Oe (blue). When excited by a 0.22 mA square-wave current, the device exhibits a clear polarity reversal upon inversion of the magnetic field direction. (f) Temperature dependence of nonreciprocity. The Josephson diode effect, quantified by the asymmetry in critical currents *I*_c_^+^ ≠ |*I*_c_^−^|, remains robust up to 86 K.

The BSCCO lattice is intrinsically anisotropic and naturally forms stacks of atomic-scale Josephson junctions—so-called intrinsic Josephson junctions (IJJs, Fig. 1a) [32]. These junctions have long been used in solid-state terahertz sources [33,34] and detectors [35]. Yet their potential for nonreciprocal superconducting transport has remained largely unexplored, with one recent study even reporting no clear diode effects in IJJs, in contrast to artificial vdW junctions [27]. This gap is striking given their atomic thickness, inherent scalability, and established role in cuprate electronics.

Here we demonstrate a high-temperature superconducting diode based on intrinsic Josephson junctions in BSCCO. By introducing controlled geometric asymmetry into stacks of intrinsic junctions, we realize pronounced supercurrent nonreciprocity persisting up to 86 K. The diode efficiency is systematically governed by the number of intrinsic junctions and reaches its maximum in the single-junction limit accessed via surface intrinsic Josephson junctions. A microscopic model based on the Lawrence-Doniach formalism reveals that geometry-induced anharmonicity in the current-phase relation, strongly amplified by the atomic-scale barriers of intrinsic junctions, underpins the observed diode effect. Leveraging the natural junction architecture of BSCCO, we further demonstrate scalable arrays containing hundreds of reproducible intrinsic Josephson diodes. These results establish intrinsic Josephson junctions as a robust, fabrication-ready platform for high-temperature superconducting diodes and open new avenues for nonreciprocal superconducting electronics based on cuprate materials.

## RESULT

### High-temperature intrinsic Josephson diode effect

The IJJ devices are created by leveraging the layered lattice structure of BSCCO crystals (Fig. 1a). Each device consists of a BSCCO stack encapsulated between top and bottom superconducting electrodes, both of which are coated with gold films (Fig. 1b). The stacked BSCCO IJJs are patterned into a wedge geometry with an inclination angle of 23°. Detailed fabrication procedures are provided in Supplementary information. The current-voltage characteristics measured at 80 K (Fig. 1c and d) show well-separated superconducting critical currents, *I*_c_^+^ and *I*_c_^−^, measured under positive and negative currents, respectively, confirming the realization of a high-temperature superconducting diode. The diode polarity can be reversed by altering the direction of the applied out-of-plane magnetic field, *H*_z_ (Fig. 1c and d). When driven by a square-wave current excitation, the device steadily switches between zero-resistance and normal-resistance states (Fig. 1e). Our temperature-dependent measurements reveal that the device operates up to 86 K (Fig. 1f), representing the highest operation temperature reported to date for superconducting diodes.

### Microscopic mechanism of intrinsic Josephson diode effect

In general, the superconducting diode effect arises from the simultaneously breaking of spatial-inversion and time-reversal symmetries [36]. In our intrinsic Josephson diode, spatial-inversion symmetry is broken by the wedge-shaped device geometry (Fig. 1b). Time-reversal symmetry is typically broken by applying a magnetic field perpendicular to the current direction [37,38]. In IJJs, however, the Josephson supercurrent flows along the out-of-plane (*z*-axis) direction of the BSCCO crystal. One would therefore expect an in-plane magnetic field—perpendicular to the supercurrent—to induce the diode effect. Contrary to this expectation, our field-orientation experiments show unambiguously that only the out-of-plane field component, parallel to the Josephson supercurrent and not conventionally linked to time-reversal symmetry breaking, governs the diode effect, while in-plane fields have negligible influence (Fig. S1). Recent studies have likewise that time-reversal symmetry can be broken in twist-angle vdW Josephson junctions under out-of-plane magnetic fields [26–29]. The microscopic origin of these Josephson diode effects, and their connection to symmetry breaking, nevertheless remains unresolved.

**Figure 2.**
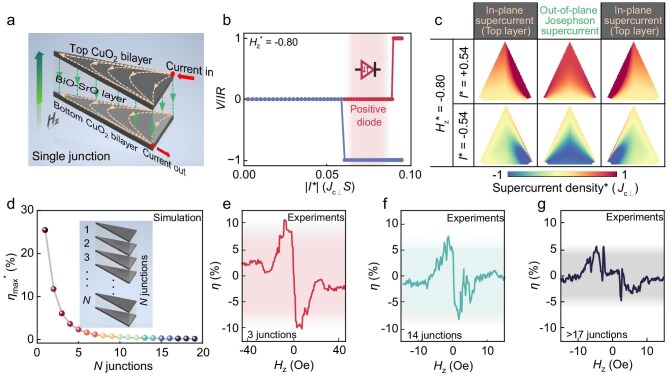
Mechanism and junction-number dependence of intrinsic Josephson diode effect. (a) Schematic of the theoretical model incorporating both in-plane supercurrents in CuO_2_ bilayers and out-of-plane interlayer Josephson supercurrents. (b) Calculated current-voltage characteristics. The critical currents exhibit strong asymmetry under the magnetic field. The magnetic field is normalized by Φ_0_/μ_0_*S*, current by ${\mathrm{\ }}{J}_{{\mathrm{c}} \bot }$*S*, and the voltage spans from 0 (superconducting state) to ±1 (resistive state). Here, Φ_0_ is the magnetic flux quantum, μ_0_ is the vacuum permeability, *S* is the junction area, and ${J}_{{\mathrm{c}} \bot }$is the Josephson critical supercurrent density. (c) Spatial distributions of supercurrent density under an out-of-plane field, highlighting broken time-reversal symmetry. (d) Calculated diode efficiency as a function of junction number. (e–g) Experimental diode efficiencies under magnetic fields, extracted from *I*_c_^+^/|*I*_c_^−^| curves in Figs S1d, S4b, and d, respectively. The number of junctions, labeled in each graph, is identified from the corresponding current-voltage characteristics in Figs S3b, S4a, and c.

To uncover the microscopic mechanism of intrinsic Josephson diodes, we model the device using the Lawrence-Doniach formalism [39], treating the interlayer Josephson current (out-of-plane) and intralayer supercurrent (in-plane) simultaneously (Fig. 2a). In this description, the supercurrent enters from one corner of the top CuO_2_ layer and exits from the opposite corner of the bottom layer (Fig. 2a). We derived a modified, self-consistent current-phase relation along each current channel (Fig. S2a–c):


(1) \begin{eqnarray*}
i = {\mathrm{sin}}{(\phi - \frac{{{\mathrm{2e}}}}{\hbar } \int {{{\mathop A\limits^{\rightarrow}}}} \cdot {{\rm d}\!\! \mathop {l}\limits^{\rightarrow}} - \kappa i)}.
\end{eqnarray*}

Here, *i* is a dimensionless current, $\frac{{{\mathrm{2}}{\rm e}}}{\hbar }\smallint \mathop A\limits^{{\mathrm{\rightarrow}}} \cdot\, {\rm d}\!\! \mathop l\limits^{{\mathrm{\rightarrow}}} $ represents the phase shift induced by the out-of-plane magnetic field, associated with time-reversal symmetry breaking [40]. *κ* is a dimensionless coefficient characterizing the anharmonicity for each current channel [41], which describes the deviation of the current-phase relation from a simple sinusoidal form (details can be found in Supplementary information). Figure 2b shows the calculated superconducting diode effect, which is consistent with the experimental observations in Fig. 1d.

We further analyzed the spatial distribution of both the in-plane intralayer supercurrent and the out-of-plane Josephson supercurrent (Fig. 2c). The results reveal that the out-of-plane magnetic field induces a nonreciprocal modulation of the in-plane supercurrent in the top and bottom CuO_2_ layers, which in turn leads to a nonreciprocal distribution of the out-of-plane Josephson supercurrent, thereby breaking time-reversal symmetry. Notably, finite values of the anharmonicity coefficient *κ* are essential for the superconducting diode effect (Fig. S2d). Our simulations indicate that when *κ* is zero, no diode effect occurs, even in the presence of time-reversal symmetry breaking (Fig. S2e, f). In conventional Josephson tunnel junctions, the anharmonicity is typically very weak [42], which results in negligible superconducting diode effects. In our modeling, the anharmonicity coefficient *κ* scales approximately as *L*^2^/*dξ*, where *L* is the length of each current channel from input to output leads, *d* is the barrier thickness of the IJJs, and *ξ* is the planar superconducting coherence length (see Supplementary information). Therefore, the anharmonicity *κ* is intrinsically determined by the device geometry and electrode configuration. The atomic-scale barrier thickness in BSCCO (*d*→0) leads to a significantly enhanced *κ*, thereby producing a prominent superconducting diode effect in IJJs.

### Junction number-dependent of the diode effect

The current-voltage characteristics of our device indicate that there are three intrinsic junctions stacked in series [43] (Fig. S3). The theoretical model described above can be extended to an *N*-IJJ stack (inset of Fig. 2d). The current-phase relationship for each current channel in the *N*-junction stacks becomes:


(2) \begin{eqnarray*}
i = {\mathrm{sin}}\left[ {\frac{{\mathrm{1}}}{{{N}}}\left( {\phi {\mathrm{\ - \ }}\frac{{{\mathrm{2e}}}}{\hbar }\int {{{\mathop A\limits^{\rightarrow}}}} \cdot {{d}}\! {\mathop l\limits^{\rightarrow} - \kappa i}} \right)} \right].
\end{eqnarray*}

This equation shows that the anharmonicity weakens as the junctions increase. In Fig. 2d, we show the calculated superconducting diode efficiency *η*, defined as *η* = (*I*_c_^+^—|*I*_c_^−^|)/(*I*_c_^+^ + |*I*_c_^−^|) × 100%, as a function of the number of stacked junctions. It clearly predicts a monotonic decay of *η* with increasing junctions.

To experimentally validate this behavior, we fabricated two more devices, which contain approximately 14 and 17 IJJs, respectively (the corresponding current-voltage characteristics are shown in Fig. S4). Figure 2e–g shows the field-dependent diode efficiency for all three devices with different numbers of junctions. The results clearly indicate that the diode efficiency is gradually enhanced in devices with fewer junctions, in agreement with our theoretical predictions. These findings further highlight the critical role of anharmonicity in enabling the superconducting diode effect in IJJs.

### Surface junctions and amplified diode effect

**Figure 3.**
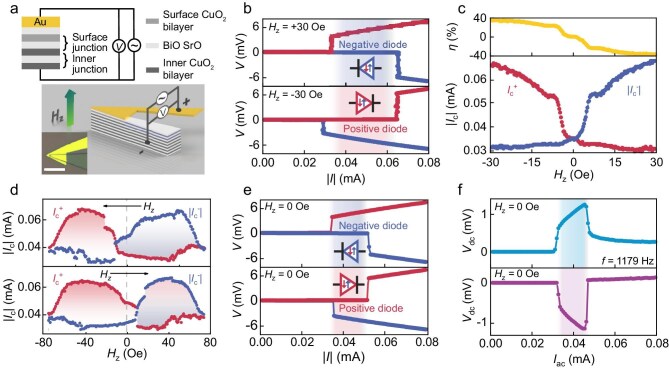
Surface intrinsic Josephson diode effect comprising a single-junction. (a) Schematic of the device structure. Superconductivity in the topmost surface CuO_2_ layer, directly contacted by the gold electrode, is slightly suppressed, resulting in a reduced critical current compared to inner junctions. The lower illustration shows the device layout. BSCCO regions unprotected by gold film have degraded. The inset displays an optical micrograph of the device. Scale bar: 30 μm. (b) Forty repeated current-voltage sweeps, demonstrating a robust and reproducible Josephson diode effect at 3 K. (c) Field-dependent critical currents and corresponding diode efficiency obtained in the low-field range. (d) Memory effect under extended magnetic fields. Reversing the field sweep direction switches the diode polarity at zero field (dashed line). (e) Current-voltage characteristics of superconducting diode effects at zero field. The diode polarity is programmed by initialization fields of *H*_z_=+75 Oe (upper panel) and *H*_z_=−75 Oe (lower panel), respectively. (f) Superconducting rectification measured under an AC excitation of 1179 Hz and at zero field.

As reducing the number of junctions increases superconducting diode efficiency (Fig. 2d), the maximum efficiency is expected in the single-junction limit. Although this situation naturally occurs in twist-angle vdW junctions [26–29], which effectively behave as single-junction diodes, controllably fabricating single-junction devices remains a considerable technical challenge. Fortunately, the surface IJJ, formed between the two uppermost CuO_2_ bilayers of the BSCCO crystal (top panel of Fig. 3a), provides a reliable route to access a single IJJ [44–46]. We fabricated a wedge-shaped surface IJJ device (bottom panel of Fig. 3a). The detailed fabrication procedures are provided in Supplementary information and Ref. [46].

As expected, the surface junction device exhibits a more pronounced superconducting diode effect (Fig. 3b). Unlike the multijunction devices, which show a relatively wide distribution of *I*_c_ (Fig. S3d), the surface intrinsic Josephson diode demonstrates excellent reliability, with sharp and reproducible switching currents under repeated operation (Fig. 3b). Notably, the diode efficiency reaches values up to 40% (Fig. 3c), substantially higher than those observed in multijunction diodes (Fig. 2e–g).

Our surface Josephson diodes display a unique memory effect that enables nonvolatile switching and reversal of the diode’s nonreciprocity. When the magnetic field is swept in the low-field region (Fig. 3c), the device exhibits an antisymmetric diode response between positive and negative current configurations, with no diode effect at zero field—that is, *I*_c_^+^(*H*_z_ = 0) *= I*_c_^−^(*H*_z_ = 0). Remarkably, after applying a larger magnetic field (|*H*_z_| > 60 Oe) to magnetize the device, a memory effect emerges (Fig. 3d): the values of *I*_c_^+^ and *I*_c_^−^ near zero field become sensitive to the device’s magnetization history. Consequently, the device displays a superconducting diode effect at zero field, as shown in Fig. 3e, with polarity determined by the direction of the initializing magnetic field. Importantly, the zero-field diode efficiency remains notable, with *η*(*H*_z_ = 0) ≈ 20%. Figure 3f demonstrates rectification of an AC signal to a DC output at zero field, demonstrating nonvolatile and reversible functionality.

The zero-field superconducting diode (or memory) effect most likely originates from trapped Abrikosov vortices in the BSCCO crystal, which break time-reversal symmetry even in the absence of an external field. The memory can be erased by warming the device above its superconducting transition temperature (Fig. S5), consistent with the behavior of trapped flux. These results highlight both the robustness and the exceptional performance of surface intrinsic Josephson diodes. Magnetic-field-induced vortices and their distribution, which can be shaped by sample geometry and defect distribution, are expected to modify the local current distribution, ultimately affecting the anharmonicity of the current-phase relation. Therefore, engineering these vortex pinning landscapes and geometric parameters presents a promising way for optimizing diode performance in future designs.

**Figure 4.**
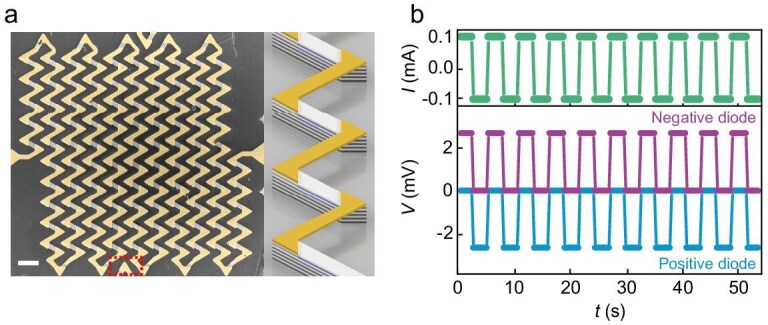
Scalable integration of Josephson diode with surface junctions. (a) Pseudocolored SEM image (left panel) of an array containing 201 serially connected surface intrinsic Josephson diodes. Scale bar: 50 μm. The right panel illustrates the schematic of the diode array configuration. Gold electrodes are shown in yellow, while degraded BSCCO regions are depicted as blue and white stacks. (b) Zero-field half-wave rectification. A square-wave current excitation, applied to the diode highlighted in the dashed box in (a), produces rectified voltage outputs at zero field for both negative and positive diode polarities.

By combining top-down fabrication with precise control of device size and geometry, we demonstrate the feasibility of producing large arrays of high-temperature intrinsic Josephson diodes. As shown in Fig. 4a, we fabricated an array of 201 serially connected surface intrinsic Josephson diodes. Transport measurements reveal highly consistent, reliable, and programmable superconducting diode behavior at zero magnetic field (Figs 4b and S6), validating both the scalability and reproducibility of high-temperature intrinsic Josephson diodes for technological applications.

## DISCUSSION AND CONCLUSION

In summary, we have established IJJs in BSCCO as a robust platform for high-temperature superconducting diodes. These devices combine all the essential properties required for practical applications: operation well above the liquid-nitrogen threshold, large diode efficiencies, nonvolatile memory functionality, and scalability to arrays containing hundreds of reproducible elements. Our combined experiments and modeling reveal that geometry-induced anharmonicity in the current-phase relation is central to the strong diode effect, with reducing the number of stacked junctions strongly enhances nonreciprocity. This framework not only explains the absence of obvious diode signals in bulk IJJs with many junctions [27], but also provides a strategy for future optimization through tailored geometries or engineered vortex pinning landscapes, including magnetic nanostructures [47] or nanoholes [48], into the IJJ architecture for a more deterministic and higher-performance Josephson diode. While the interface quality required for high-coherence quantum applications remains beyond the reach of the present methodology [49], our platform is nonetheless relevant for circuit-level functionalities, such as nonreciprocal signal routing [50] and rectification [3,4].

## METHODS

The fabrication procedures of high-temperature superconducting intrinsic Josephson junction device, surface intrinsic Josephson junction device, transport measurements, and Lawrence-Doniach simulation are shown in Supplementary data.

## Supplementary Material

nwag285_Supplemental_Files
