# Supplementary material for: Scalable high-temperature superconducting diodes enabled by intrinsic Josephson junctions
Source: Natl Sci Rev. 2026 May 22;13(14):nwag285. doi: 10.1093/nsr/nwag285 (PMC13348245; doi:10.1093/nsr/nwag285)
Supplement: nwag285_Supplemental_Files [file nwag285_supplemental_files.zip › NSR_MS-2025-2992-supporting/NSR_MS-2025-2992-supporting.docx]

Supplementary Data for

**Scalable high-temperature superconducting diodes enabled by intrinsic Josephson junctions**

Zihan Wei^1,2,†^, Youkai Qiao^3,†^, Yang-Yang Lyu^1,†,*^, Da Wang^3,*^, Tianyu Li^1^, Leonardo Rodrigues Cadorim^4^, Ping Zhang^1,2^, Wen-Cheng Yue^1^, Dingding Li^1,2^, Ziyu Song^1^, Zixi Wang^1^, Yunfan Wang^1^, Milorad V. Milošević^4^, Yong-Lei Wang^1,2,5,*^, Huabing Wang^1,2,*^, Peiheng Wu^1,2^

^1^*Research Institute of Superconductor Electronics (RISE) & Key Laboratory of Optoelectronic Devices and Systems with Extreme Performances of MOE, School of Electronic Science and Engineering, Nanjing University, Nanjing 210023, China*

^2^*Purple Mountain Laboratories, Nanjing 211111, China*

^3^*National Laboratory of Solid State Microstructures & School of Physics, Nanjing University, Nanjing 210023, China*

^4^*COMMIT, Department of Physics, University of Antwerp, Antwerp 2000, Belgium*

^5^*State Key Laboratory of Spintronics Devices and Technologies, Nanjing University, Nanjing 210093, China*

yylyu@nju.edu.cn, dawang@nju.edu.cn, yongleiwang@nju.edu.cn, hbwang@nju.edu.cn

**1. Fabrication of intrinsic Josephson junction device**

BSCCO single crystals were grown using the floating-zone method. IJJ devices were fabricated via a double-sided patterning technique^43^ (Fig. S7). Crystals were mounted on sapphire substrates using epoxy, with the *c*-axis oriented perpendicular to the substrate. A 50-nm-thick gold film was immediately deposited onto the freshly cleaved surface (a). A wedge-shaped mesa was subsequently defined using standard photolithography and ion milling, producing a structure with a typical thickness of ~300 nm (b). Additional ion milling was applied to etch step structures at the wedge, with an etching depth slightly less than 300 nm, to define one side of the device electrode (c). The entire surface was then encapsulated in epoxy, and a second sapphire substrate was applied on top (d). After curing at elevated temperature, the sample was mechanically split (e) and flipped onto the new substrate (f). A second 50-nm-thick gold layer was deposited on the newly exposed surface, and the opposite electrode was patterned through a third round of photolithography (g). The final number of junctions in each device was controlled by adjusting the final etching depth of ion milling (h).

**2. Fabrication of surface intrinsic Josephson junction device**

The top BSCCO electrode in conventional IJJ devices was replaced by a gold electrode, deposited via *in-situ* high-vacuum evaporation at room temperature (Fig. S8). A small piece of BSCCO crystal was mounted onto a sapphire substrate using epoxy, with its 𝑐-axis perpendicular to the surface. The sample was then transferred into a high-vacuum evaporator, where a piece of Scotch tape was attached to the shutter to enable *in-situ* cleavage of the crystal. Once the chamber was preheated to the desired temperature, the shutter was opened to cleave the crystal, immediately followed by the deposition of a 200-nm-thick gold film (a) at a deposition rate of ~18 nm/s. A smooth region on the cleaved surface was selected, and wedge-shaped BSCCO stacks were patterned using conventional photolithography and ion milling (b). A SiO*_x_* insulating layer was subsequently deposited and lifted off in acetone, forming an insulating layer with thickness matched to the surrounding crystal (c). A 100-nm-thick gold layer was deposited and patterned using photolithography (d). Finally, wet etching (KI/I_2_) was used to define electrodes while minimizing damage to the underlying BSCCO layers (e). The surface IJJs are located in the wedge tip region, characterized by a top angle of 23 degrees.

**3. Transport measurements**

Transport measurements were performed in cryogen-free cryostats equipped with three-axis superconducting magnets (Cryomagnetics, Inc.) for IJJ devices and a GM refrigerator (Sumitomo Corp.) with a custom-built coil for surface IJJ devices. DC measurements utilized a homemade current source and a low-noise voltage amplifier, powered by a 12 V lead-acid battery to suppress noise. Data acquisition was controlled via an NI PCI-6221 data acquisition card. For AC measurements, a Keithley 6221 current source and 2182A nanovoltmeter were employed. IJJ devices were configured for four-terminal measurements, while surface IJJ devices used a three-terminal setup, with the top gold layer serving both current and voltage leads. The contact resistance between the gold electrode and the uppermost CuO_2_ layer was typically lower than 1 Ω.

**4. Lawrence-Doniach simulation**

To model the behavior of layered superconductors, we adopted the Lawrence–Doniach framework, which extends beyond the conventional anisotropic Ginzburg–Landau theory by treating the inter-layer coupling as Josephson tunneling. In this model, the in-plane supercurrent density within the *n*-th layer is governed by the London equation:

$${\vec{\text{J}}}_{\text{∥}}^{\text{n}}\text{ = }\text{ρ}_{\text{s}}\left( \frac{\text{ℏ}}{\text{2}\text{e}}\text{∇}\text{ϕ}_{\text{n}}\text{-}\vec{\text{A}} \right)\text{ }\text{ }\text{ }\text{ }\text{ (}\text{3}\text{)}$$

while the Josephson supercurrent density between the *n*-th and (*n*+1)-th layers is described by the Josephson relation:

$$\text{J}_{\text{⊥}}^{\text{n}\text{+1, }\text{n}}\text{ =}{\text{ }\text{J}}_{\text{c}\text{⊥}}\sin\left( \text{ϕ}_{\text{n}\text{+1}} \text{- }\text{ϕ}_{\text{n}} \right)\text{ }\text{ }\text{ }\text{ }\text{ (}\text{4}\text{)}$$

where $\text{ρ}_{\text{s}}$ is the in-plane superfluid density, $\text{J}_{\text{c}\text{⊥}}$ is the critical Josephson current density, $\text{ϕ}_{\text{n}}$ is the phase of the *n*-th layer, and $\vec{\text{A}}$ is the vector potential.

**5. Temperature dependence of intrinsic Josephson diode**

IJJ devices exhibit a superconducting transition temperature of approximately 90 K (Fig. S3a). At a low temperature of 3 K, the distributions of the statistically evaluated critical currents *I*_c_^+^ and |*I*_c_^-^|, obtained from 1000 repeated current-voltage curves, are relatively broad (Fig. S3c). Under zero magnetic field, the distributions of *I*_c_^+^ and *I*_c_^-^ nearly coincide, indicating the absence of a superconducting diode effect. An out-of-plane magnetic field (*H*_z_) leads to a clear separation of *I*_c_^+^ and *I*_c_^-^ into two peaks, revealing the emergence of the superconducting diode effect. The partial overlap of the *I*_c_^+^ and |*I*_c_^-^| distributions limits switching accuracy, consistent with behaviors previously reported in Josephson diode based on twist-angle BSCCO flakes^27^. Temperature-dependent measurements (Fig. S3d) show a gradual narrowing of the overlap region as temperature increases, with full separation above 65 K, thereby enabling reliable high-temperature operation of the Josephson diodes. Due to microfabrication processes such as ion milling, slight degradation, structural misalignment, or local inhomogeneity may occur in the BSCCO crystal, which can introduce additional weak or spatially nonuniform junction contributions outside the main current path. A prominent example is observed in Fig. 1c and 1d, where a distinct current kink appears near 0.22 mA. Detailed analysis reveals that the voltage increment associated with this kink matches the characteristic voltage of a single Josephson junction, as corroborated by Fig. S3e.

**6. Magnetic field orientation dependence of intrinsic Josephson diode**

To investigate the dependence of the intrinsic Josephson diode effect on magnetic field orientation, we characterized our device under three-dimensional magnetic field configurations (Fig. S1). Figure S1b shows the critical currents measured under magnetic fields *H*_r_ applied at three different directions (*θ* = 30° and *φ* = 0°, 45° and 90°). Asymmetric values of *I*_c_^+^ and *I*_c_^-^ were observed in each case within [-20 Oe, +20 Oe]. The near-complete overlap of the three *I*_c_-*H*_r_ curves indicates a negligible influence from the in-plane field components (*H*_x_/*H*_y_). To clarify the role of the out-of-plane field component, we measured the critical currents in the *x*–*z* field plane (*φ* = 0°) while varying *θ* (Fig. S1c). Despite changes in *θ* from 0° to 45°, the overall shapes of the *I*_c_-*H*_r_ curves remained essentially unchanged. When plotted as a function of the out-of-plane field component (Fig. S1d), all curves collapsed onto a single trace, confirming that the out-of-plane field is the dominant component driving the intrinsic Josephson diode effect. This behavior is consistent with the two-dimensional nature of the layered BSCCO crystals.

**7. Transport properties of surface intrinsic Josephson diode**

Surface IJJs exhibit weaker interlayer coupling compared to inner IJJs, resulting in lower critical currents (Fig. S9b) and reduced superconducting transition temperatures (Fig. S9a). The moderate degradation in our surface junction devices arises from fabrication-induced disorder^1-4^, particularly oxygen loss and structural reconstruction at the interface during the interval between cleavage and metal deposition, which can be effectively suppressed by high-rate *in-situ* evaporation under ultra-high vacuum^5,6^. This reduced superconductivity enables selective probing of surface IJJ dynamics at low bias. Over a broad current range, the device displays typical current-voltage characteristics of IJJs (Fig. S9b). At low currents, a clear hysteresis—indicative of a single Josephson junction—is observed (inset of Fig. S9b).

When driven by a square-wave current with amplitude between *I*_c_^+^ and *|I*_c_^-^|, the device maintained stable diode operation for over 10,000 cycles (Fig. S9c), demonstrating both robust performance and excellent rectification behavior. Furthermore, the zero-field superconducting diode effect was retained following exposure to large field magnetization. The onset threshold for such field-programmed diode behavior was determined by systematically varying the applied *H*_z_ field (Fig. S9d).

**8. Simulation model of intrinsic Josephson junction**

We first consider a current channel within a single IJJ (Fig. S2a and S2b). The supercurrent enters from the top-right and exits from the bottom-left corner. Due to the non-uniformity of in-plane current density, the cross-sectional width $\text{b}_{\text{l}}$ of the channel varies along the propagation distance $\text{l}$. Neglecting the vector potential for simplicity, we assume that the phase difference $\text{δ}_{\text{l}}$ between the two superconducting layers remains constant, denoted as $\text{δ}$, a simplifying approximation validated by numerical calculations (Fig. S10). The total current *I* flowing through the channel is the sum of vertical supercurrents:

*I* = $\int\text{J}_{\text{c}\text{⊥}}\sin\text{δ}\text{ }\text{b}_{\text{l}}\text{dl}\text{ }\text{= }\text{J}_{\text{c}\text{⊥}}\text{S}\sin\text{δ}\text{ }\text{ }\text{ }\text{ }\text{ (}\text{5)}$

where *S* $\text{= }\int\text{b}_{\text{l}}\text{dl}$ is the total area of each layer.

On the other hand, the total current can also be calculated by summing the in-plane supercurrents in both layers:

$$\frac{\text{I}}{\text{b}_{\text{l}}\text{d}}\text{ = }\frac{\text{ℏ}}{\text{2}\text{e}}\text{ρ}_{\text{s}}\left( \text{∇}\text{ϕ}_{\text{1}}\text{ + }\text{∇}\text{ϕ}_{\text{2}} \right)\text{ }\text{ }\text{ (6)}$$

where $\text{ϕ}_{\text{1}}\text{,}{\text{ }\text{ϕ}}_{\text{2}}$ are phases for the two layers, $\text{d}$ is the vertical inter-layer displacement. Taking a line integral along the path $l$, we obtain

$$\frac{\text{I}}{\text{d}}\int\frac{\text{dl}}{\text{b}_{\text{l}}}\text{ = }\frac{\text{ℏ}}{\text{2}\text{e}}\text{ρ}_{\text{s}}\left( \text{ϕ}\text{ + }\text{ϕ}\text{ - }\text{δ}\text{ -}\text{δ} \right)\text{ = }\frac{\text{ℏ}}{\text{e}}\text{ρ}_{\text{s}}\left( \text{ϕ }\text{- }\text{δ} \right)\text{ }\text{ }\text{ (7)}$$

from which we obtain$\text{δ}\text{ = }\text{ϕ }\text{- }\left( \frac{\text{e}}{\text{ℏ}\text{d}\text{ρ}_{\text{s}}}\int_{\text{l}} \text{b}_{\text{l}}^{\text{-1}} \right)\text{I}$. Substituting this into Eq. (5) gives the current-phase relation:

*i* = $\sin\left( \text{ϕ}\text{ - }\text{κi} \right)\text{,}\text{ }\text{ }\text{ }\text{ (}\text{8)}$

where $\text{i}\text{ = }\frac{\text{I}}{\text{J}_{\text{c}\text{⊥}}\text{S}}$ is the dimensionless current and $\text{κ}$ denotes a dimensionless anharmonicity parameter defined as:

*κ*$\text{ = }\frac{\text{e}}{\text{ℏ}}\frac{\text{J}_{\text{c}\text{⊥}}\text{S}}{\text{d}\text{ρ}_{\text{s}}}\text{ }\int_{\text{l}} \frac{\text{dl}}{\text{b}_{\text{l}}}\text{ = }\frac{\text{1}}{\text{3}\sqrt{\text{3}}}\frac{\text{L}^{\text{2}}}{\text{d}\text{ξ}_{\text{∥}}}\frac{\text{J}_{\text{c}\text{⊥}}}{\text{J}_{\text{c}\text{∥}}}\text{⟨}\text{b}_{\text{l}}\text{⟩⟨}\text{b}_{\text{l}}^{\text{-1}}\text{⟩}\text{ }\text{ }\text{ }\text{ }\text{ }\text{ (}\text{9)}$

where we use the Ginzburg–Landau expression for the in-plane critical current density $\text{J}_{\text{c}\text{∥}}\text{ = }\frac{\text{1}}{\text{3}\sqrt{\text{3}}}\frac{\text{ℏ}}{\text{e}}\frac{\text{ρ}_{\text{s}}}{\text{ξ}_{\text{∥}}}$, $\text{L}$ is the channel length, $\left\langle\text{b}_{\text{l}} \right\rangle$ and $\langle\text{b}_{\text{l}}^{\text{-1}}\rangle$ are average values of $\text{b}_{\text{l}}$ and $\text{b}_{\text{l}}^{\text{-1}}$ along the channel.

In the presence of a vertical magnetic field, the current-phase relationship within a current channel can be generalized by replacing the phase difference $\text{ϕ}$ with its gauge-invariant form $\left( \text{ϕ}\text{-}\frac{\text{2}\text{e}}{\text{ℏ}}\int\vec{\text{A}}\text{⋅}\text{d}\vec{\text{l}} \right)$:

*i*$\text{ = }\sin\left( \text{ϕ}\text{ - }\frac{\text{2}\text{e}}{\text{ℏ}}\int\vec{\text{A}}\text{⋅}\text{d}\vec{\text{l}} \text{- }\text{κi} \right)\text{ }\text{ }\text{ }\text{ }\text{ }\text{ (}\text{10)}$

It gives anharmonic current-phase relationship for nonzero $\text{κ}$. The anharmonicity is enhanced by enlarging $\text{κ}$ (Fig. S2c). From the definition of $\text{κ}$, it is evident that it can be significantly enhanced by increasing the factor $\frac{\text{L}^{\text{2}}}{\text{d}\text{ξ}_{\text{∥}}}$.

The total current $\text{I}_{\text{tot}}$ is the sum of all the channels (denoted by $\text{k}$) $\text{I}_{\text{tot}}\text{(}\text{ϕ}\text{) = }\sum_{\text{k}} \text{i}_{\text{k}}\text{(}\text{ϕ}\text{)}$. By varying the phase difference $\text{ϕ}$ between the two leads, the maximal and minimal values of the total current $\text{I}_{\text{tot}}\text{(}\text{ϕ}\text{)}$ define the critical current *I*_c_^+^ and *I*_c_^-^, respectively. In general, *I*_tot_(*ϕ*) = $\sum_{\text{n}} \text{I}_{\text{cn}}$sin(*nϕ* + *δ_n_*). As shown in Fig. S2d, the introduction of anharmonicity leads to direction-dependent asymmetry in the critical current under a magnetic field. The critical current difference that reverses with the magnetic field polarity is a direct manifestation of the diode effect. If all the anharmonic terms with *n* > 1 are zero, we must always have |*I*_c_^+^| = |*I*_c_^-^|, hence, without the diode effect (Fig. S2e), despite the time-reversal symmetry breaking induced by magnetic fields (Fig. S2f). Therefore, anharmonicity is a necessary condition for the Josephson diode effect. This mechanism is further visualized in Video S1, which demonstrates the dynamic in-plane and interlayer current density distributions under negative magnetic field. The magnetic field modulation of current flow, influenced by anharmonicity, leads to *I*_c_^+^ > |*I*_c_^-^|. In contrast, Video S2 shows that without a magnetic field, the current remains symmetric.

**9. Extension to multi-junctions**

The model generalizes naturally to an *N*-junction stack. Assuming that the vertical phase differences are uniform 𝛿 across all junctions, the in-plane current in middle layers are all zero except top and bottom layers, due to the current conservation. Therefore, phases of different layers from top to bottom are: $\text{ϕ}_{\text{l}}\text{, }\text{ϕ}_{\text{l}}\text{ + }\text{δ}\text{, }\text{ϕ}_{\text{l}}\text{ + 2}\text{δ}\text{,}\text{ }\text{⋯}\text{, }\text{ϕ}_{\text{l}}\text{ + }\text{Nδ}$. Following a similar derivation to the single junction case, the current-phase relation within a current channel generalizes to:

*i* $\text{= }\sin\left[ \frac{\text{1}}{\text{N}}\left( \text{ϕ }\text{- }\frac{\text{2}\text{e}}{\text{ℏ}}\int\vec{\text{A}}\text{⋅}\text{d}\vec{\text{l}}\text{-}\text{κi} \right) \right]\text{ }\text{ }\text{ }\text{ }\text{ }\text{ (}\text{11)}$

Thus, increasing the number of junctions *N* reduces the anharmonicity, which explains the experimentally observed suppression of Josephson diode effect in devices with more junctions.

**10. Numerical simulations of triangular-shaped device**

We numerically simulated the Lawrence-Doniach model for a triangular-shaped *N*-junction stack. Ideally, both current and phase distributions should be determined by minimizing the total free energy. However, for the complex geometry of triangular multi-junctions, full finite-element simulations are computationally demanding. At this stage, we therefore adopted a simplified model, assuming that the current follows along multiple bilayer channels (Fig. 2a). The device can be regarded as a parallel connection of these current channels, all sharing a common input phase (set to zero) and output phase $\text{ϕ}$.

For the numerical calculation, each in-plane path is discretized into $\text{M}$ slices with length $\text{Δ = }\text{L}\text{/}\text{M}$. The phases of the two layers are denoted as $\text{ϕ}_{\text{n}\text{, }\text{l}}\text{ (}\text{n }\text{= 1, 2,}\text{ }\text{⋯}\text{N}\text{+1}\text{; }\text{l}\text{ = 0, 1, 2,}\text{ }\text{⋯}\text{M}\text{)}$, respectively. Then, the in-plane current can be expressed as:

$$\frac{\text{I}_{\text{∥}\text{, }\text{n}\text{, }\text{l}\text{→}\text{l}\text{+1}}}{\text{d}\text{b}_{\text{l}}}\text{ = }\frac{\text{ℏ}}{\text{2}\text{e}}\text{ρ}_{\text{s}}\frac{\text{ϕ}_{\text{n}\text{, }\text{l}\text{+1 }} \text{- }\text{ϕ}_{\text{n}\text{, }\text{l}}\text{-}\frac{\text{2}\text{e}}{\text{ℏ}}{\vec{\text{A}}}_{\text{l}}\text{⋅}{\vec{\text{Δ}}}_{\text{l}}}{\text{Δ}}\text{ }\text{ }\text{ }\text{ }\text{ (12)}$$

and the vertical current is:

$$\text{I}_{\text{⊥}\text{, }\text{n}\text{→}\text{n}\text{+1, }\text{l}}\text{ =}{\text{ }\text{J}}_{\text{c}}\text{b}_{\text{l}}\text{Δ}\sin\left( \text{ϕ}_{\text{n}\text{+1, }\text{l}} \text{-}{\text{ }\text{ϕ}}_{\text{n}\text{, }\text{l}} \right)\text{ }\text{ }\text{ }\text{ (13)}$$

In practice, we fix $\text{ϕ}_{\text{1,0}}\text{ = 0}$, and choose $\text{ϕ}_{\text{n}\text{, 0}}\text{(}\text{n }\text{> 1)}$ as variational parameters to minimize an objective function *O*$\left( \left\{ \text{ϕ}_{\text{n}\text{, 0}} \right\} \right)\text{ = }\left| \text{I}_{\text{∥}\text{, 1, }\text{M}\text{→}\text{M}\text{+1}} \right|\text{ + }\left| \text{I}_{\text{∥}\text{, 2, }\text{M}\text{→}\text{M}\text{+1}} \right|\text{ +}\text{⋯}\text{+ }\left| \text{I}_{\text{∥}\text{, }\text{N}\text{, }\text{M}\text{→}\text{M}\text{+1}} \right|$. After optimization, $\text{ϕ}\text{ =}{\text{ }\text{ϕ}}_{\text{N}\text{+1}\text{, }\text{M}}$ is extracted to obtain the current-phase relation $\text{I}\text{(}\text{ϕ}\text{)}$ for this channel.

We have performed numerical calculations for $\text{N}\text{ = }\text{1, }\text{2, 3}$, respectively. We found the results of current-phase relation for each channel $\text{I}_{\text{N}\text{, }\text{k}}\text{(}\text{ϕ}\text{)}$ can be described by Eq. (11) quite well (Fig. S10). Based on this observation, we only need to obtain the anharmonicity parameter $\kappa$ for each channel for $\text{N}\text{ = }\text{1}$, and then apply it to any $\text{N}\text{ > }\text{1}$ following Eq. (13) directly. In this way, we can predict the diode coefficient for all multi-junction stacks.

**Supplementary Video 1.** Spatial distribution of supercurrent densities under a finite *H*_z_ field. The top six color maps display the spatial distributions of in-plane and Josephson supercurrents under opposite bias currents (*I**=±0.54) with a fixed out-of-plane magnetic field (*H*_z_*=-0.80). The bottom graph shows the device resistance as a function of bias current.

**Supplementary Video 2.** Spatial distribution of supercurrent densities at zero field. The top six color maps display the spatial distributions of in-plane and Josephson supercurrents under opposite bias currents (*I**=±0.54) at zero field (*H*_z_*=0). The bottom graph shows the device resistance as a function of bias current.

**REFERENCES**

1. Martini M, Lee Y, Confalone T *et* *al*. Twisted cuprate van der Waals heterostructures with controlled Josephson coupling. *Mater*. *Today* 2023; **67**: 106-112.
2. Zhao S Y F, Cui X, Volkov P A *et* *al*. Time-reversal symmetry breaking superconductivity between twisted cuprate superconductors. *Science* 2023; **382**: 1422-1427.
3. Confalone T, Sardo F L, Montemurro D *et* *al*. Preserving the Josephson coupling of twisted cuprate junctions via tailored silicon nitride circuits boards. *Small* 2025; **21**: e06520.
4. Lau C N, Bockrath M W, Mak K F, Zhang F. Reproducibility in the fabrication and physics of Moiré materials. *Nature* 2022; **602**: 41-50.
5. Lee Y, Martini M, Confalone T *et* *al*. Encapsulating high-temperature superconducting twisted van der Waals heterostructures blocks detrimental effects of disorder. *Adv. Mater.* 2023; **35**: 2209135.
6. Wei Z, Du H, Li D *et* *al*. Tailoring Bi_2_Sr_2_CaCu_2_O_8+δ_ surface Josephson junctions. *Appl Phys Lett* 2023; **122**: 112601.

**
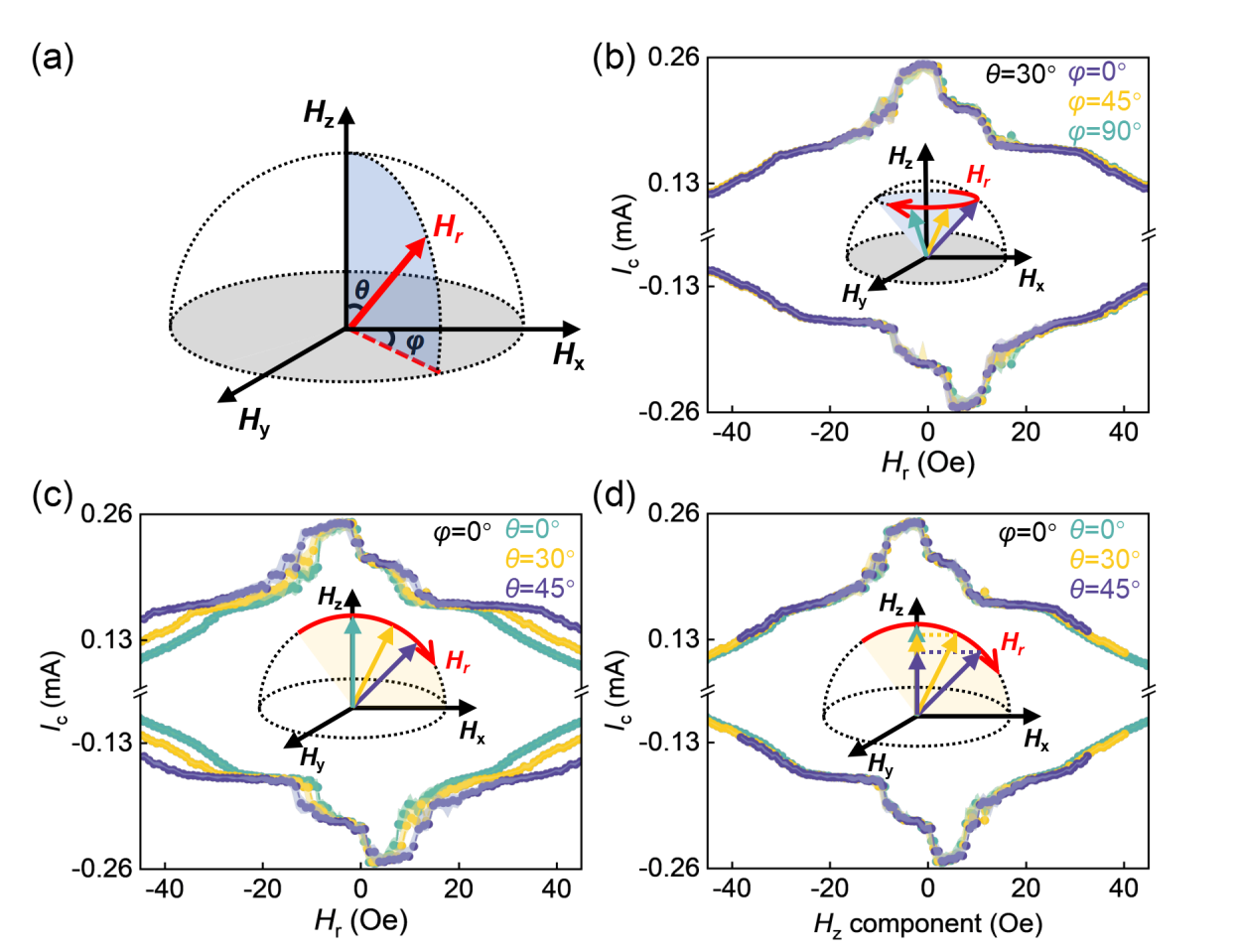
**

**Supplementary Figure 1.** Angular dependence of critical currents at 80 K. (a) Definition of the spherical coordinate system used to describe magnetic field orientation, with polar (*θ*) and azimuthal (*φ*) angles. (b) Critical currents measured under three magnetic field directions: *θ=*30^o^, *φ=*0^o^, 45^o^, and 90^o^, respectively. Colored curves correspond to different field directions indicated by arrows in the inset. Dots represent the averaged critical currents from 100 repeated measurements. (c) Critical currents measured under magnetic fields confined to the *x*–*z* plane (*φ=*0^o^), for *θ=*0^o^, 30^o^, and 45^o^. (d) Critical currents plotted as a function of the out-of-plane field component (*H*_z_), extracted from (c). The near-complete overlap of three curves demonstrates that the diode effect is primarily governed by the out-of-plane field component, with negligible contribution from the in-plane field.


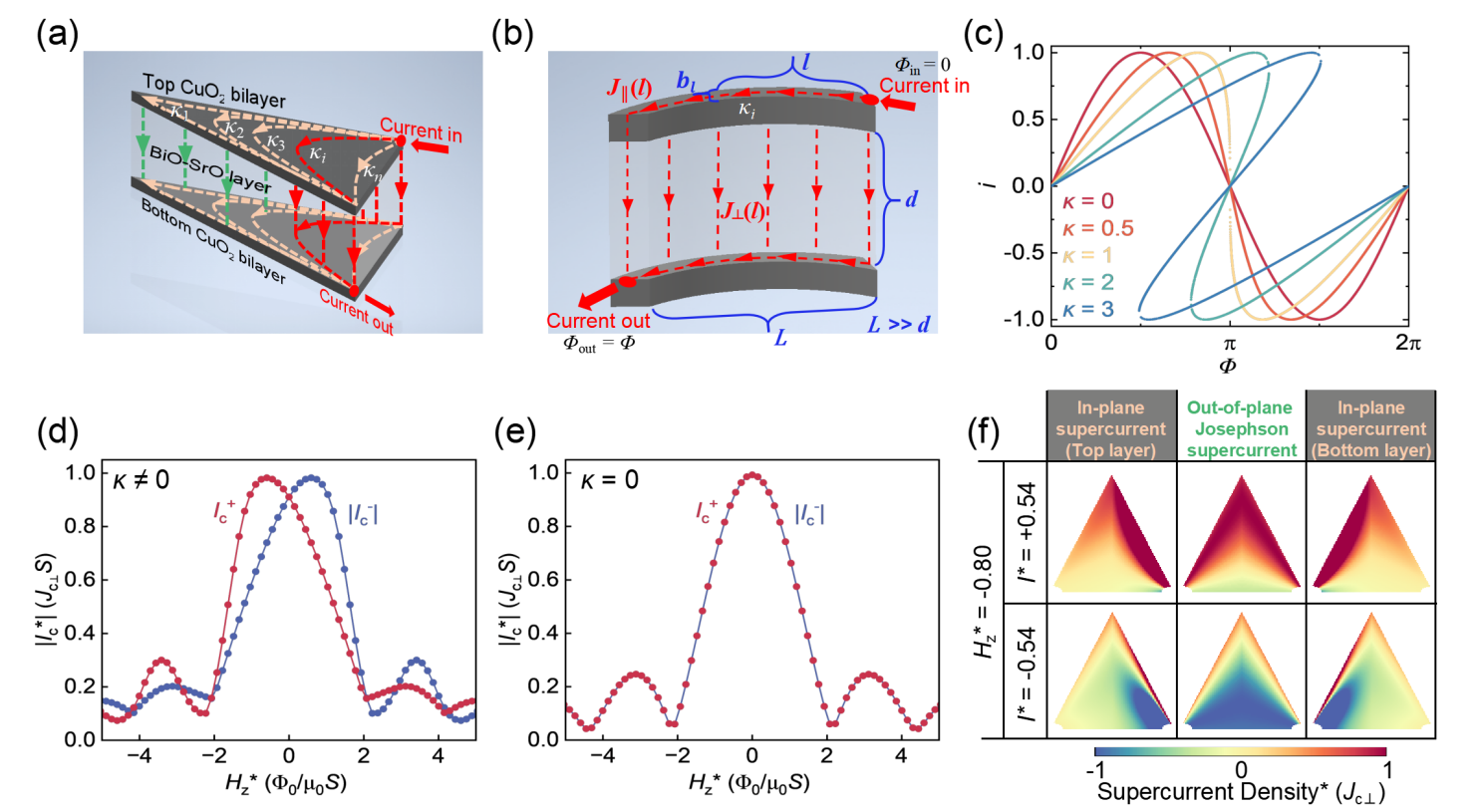


**Supplementary Figure 2.** The current channel model and role of anharmonicity in nonreciprocity. (a) Schematic of the theoretical framework, accounting for in-plane supercurrents in the superconducting CuO_2_ bilayers (orange arrows) and the out-of-plane interlayer Josephson supercurrents (green arrows). A representative current channel is outlined with a dashed red contour, as shown in (b). The model comprises multiple current channels, each characterized by anharmonicity parameters *κ*_1_, *κ*_2_…*κ_n_*, with the total current being the sum of the contributions from all individual channels. (b) Schematic illustration of a current channel within an IJJ. (c) Anharmonic current-phase relation for the current channel, modulated by tuning the anharmonicity parameter *κ*. (d) Theoretical field-dependent critical currents for *κ*≠0. Two curves exhibit clear nonreciprocity when a finite anharmonicity parameter *κ* is introduced. (e) Theoretical field-dependent critical currents for *κ*=0. The absence of Josephson diode behavior (perfect overlap of the critical currents for positive and negative bias) confirms that a nonzero *κ* is essential for Josephson diodes. (f) Spatial distributions of supercurrent densities with *κ*=0. Similar to the results in Fig. 2c, clear differences between positive and negative bias are seen, indicating time-reversal symmetry breaking under a finite magnetic field.


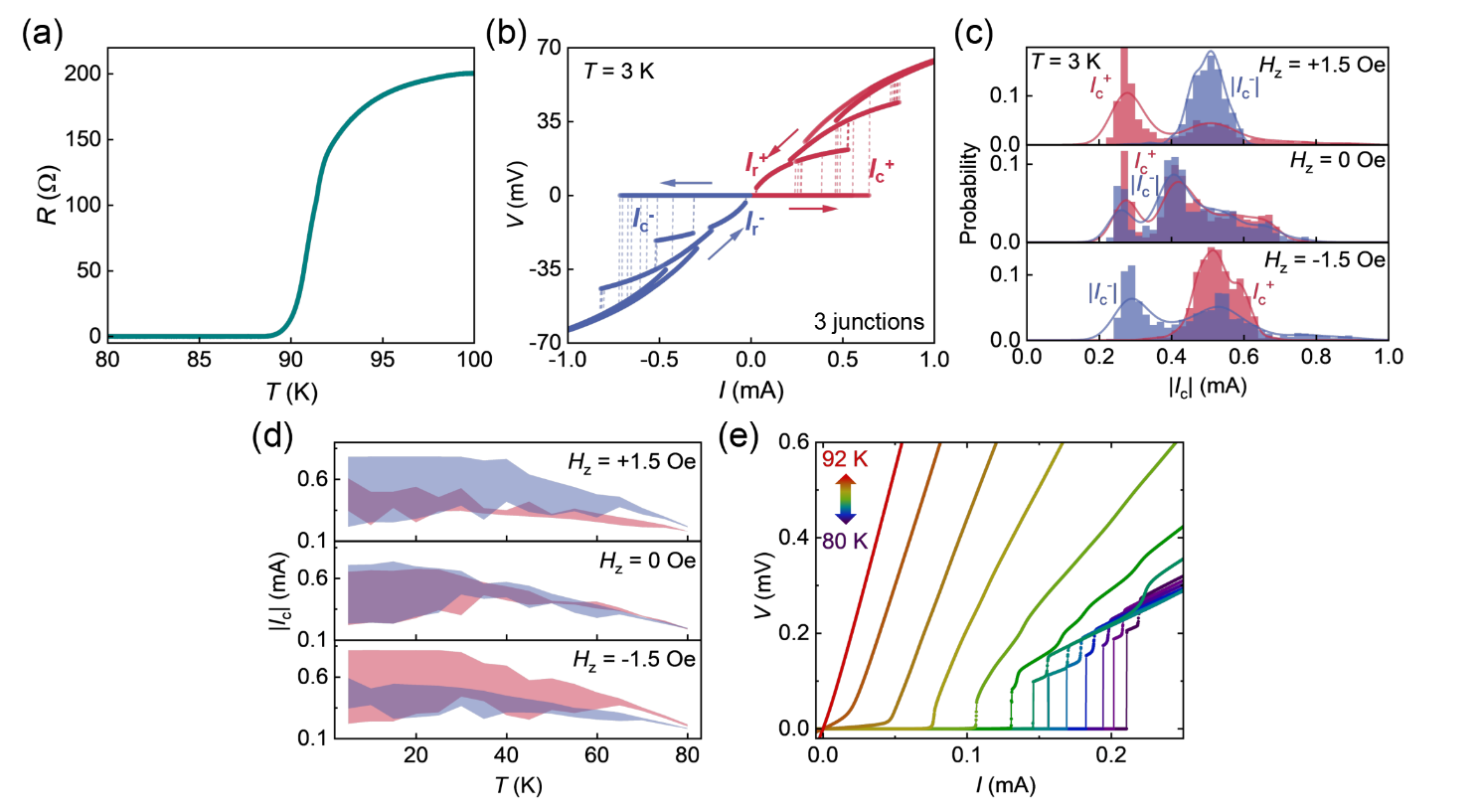
**Supplementary Figure 3.** Transport properties of the intrinsic Josephson junction device. (a) Temperature dependence of resistance, which is measured under a 10 μA bias by subtracting voltages from opposite current polarities to eliminate offset. The superconducting transition temperature of the device is approximately 90 K. (b) Current-voltage characteristics with ten repeated sweeps under cyclic bias current. Arrows denote sweep direction. Red and blue curves correspond to positive and negative current branches, respectively. The dashed line indicates the critical currents. The three voltage branches indicate the presence of three IJJs^43^. (c) Statistical distributions of critical currents. Each panel shows the probability distribution of *I*_c_ obtained from 1,000 repeated current-voltage measurements under different magnetic fields. (d) Temperature dependence of critical current distributions**.** Shaded areas indicate the full range of critical current distributions (positive: red, negative: blue) as a function of temperature, obtained from 100 repeated current-voltage measurements. (e) Temperature-dependent current-voltage characteristics from 80 K to 92 K at zero field.

**
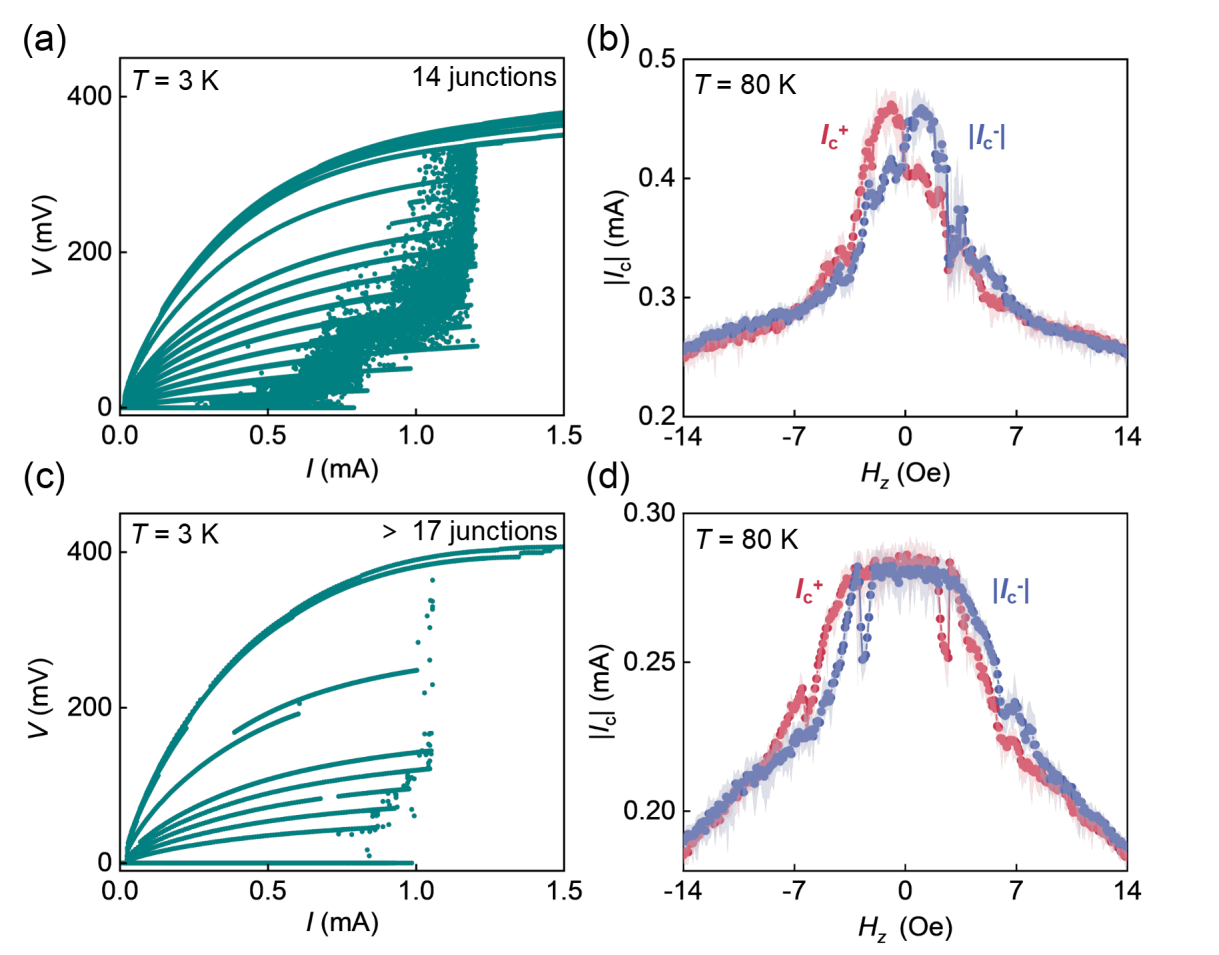
**

**Supplementary Figure 4.** Multicycle transport measurements of two additional intrinsic Josephson junction devices. (a and c) Current-voltage characteristics from 1,000 repeated measurements. The number of voltage branches reflects the number of junctions in each device. (b and d) Magnetic field dependence of critical currents corresponding to devices in (a) and (c), respectively. Solid dots indicate averaged values from 100 measurements, and shaded regions indicate distribution widths.


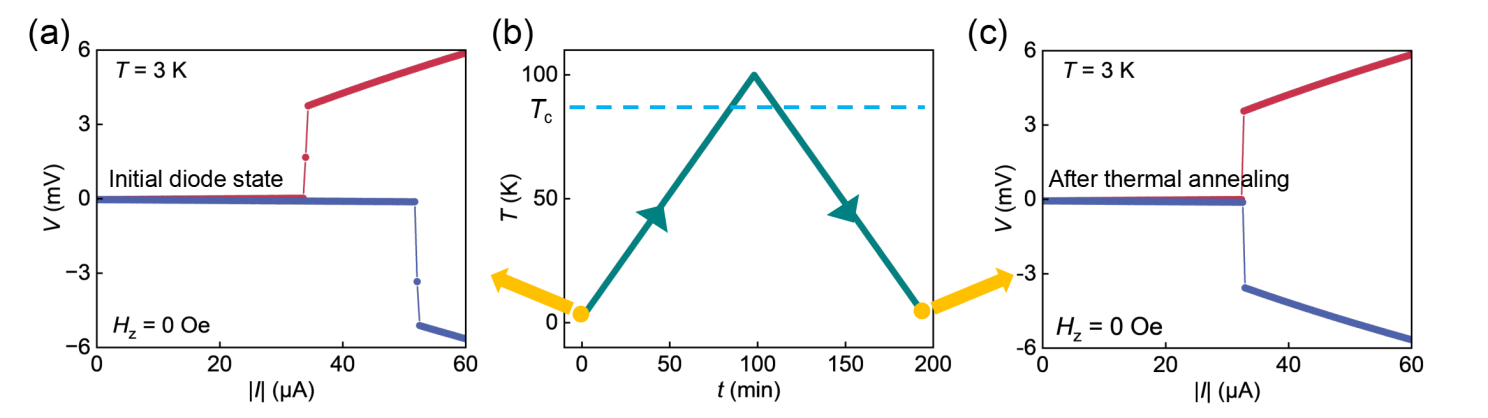


**Supplementary Figure 5.** Thermal erasure of the zero-field Josephson diode effect. (a) Stable diode behavior observed at zero magnetic field. (b) Schematic of thermal cycling: initial measurement in (a) at 3 K, heating up to 100 K, and re-cooling measurement in (c) at 3 K. The blue dashed line indicates the superconducting transition temperature (*T*_c_) of BSCCO crystals. (c) Post-annealing current-voltage characteristics. After the thermal cycling, the nonreciprocity vanishes at zero field, indicating that the zero-field Josephson diode state is erased by thermal treatment.


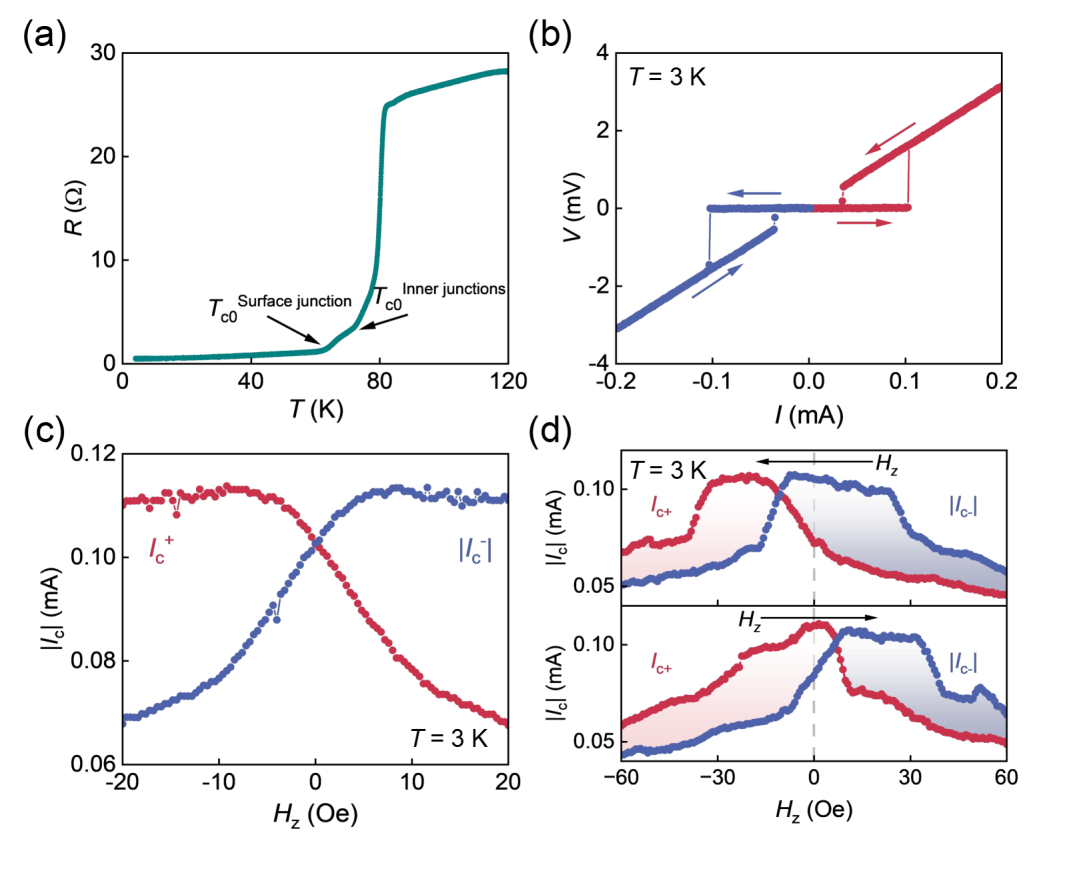


**Supplementary Figure 6.** Transport properties of a selected surface intrinsic Josephson diode from the large array. (a) Temperature dependence of resistance. The bias current is 10 μA, and voltages were obtained by subtracting responses from positive and negative currents to eliminate voltage offsets. (b) Current-voltage characteristics at zero field. Arrows indicate the sweep direction of bias currents. (c) Magnetic field dependence of critical currents in the low-field region. The critical currents for reversed bias are symmetric at zero field. (d) Magnetic field dependence of critical currents under high-field sweep conditions. The results reveal history-dependent critical currents (*I*_c_^+^ and |*I*_c_^-^|), with the zero-field difference highlighting the memory effect.


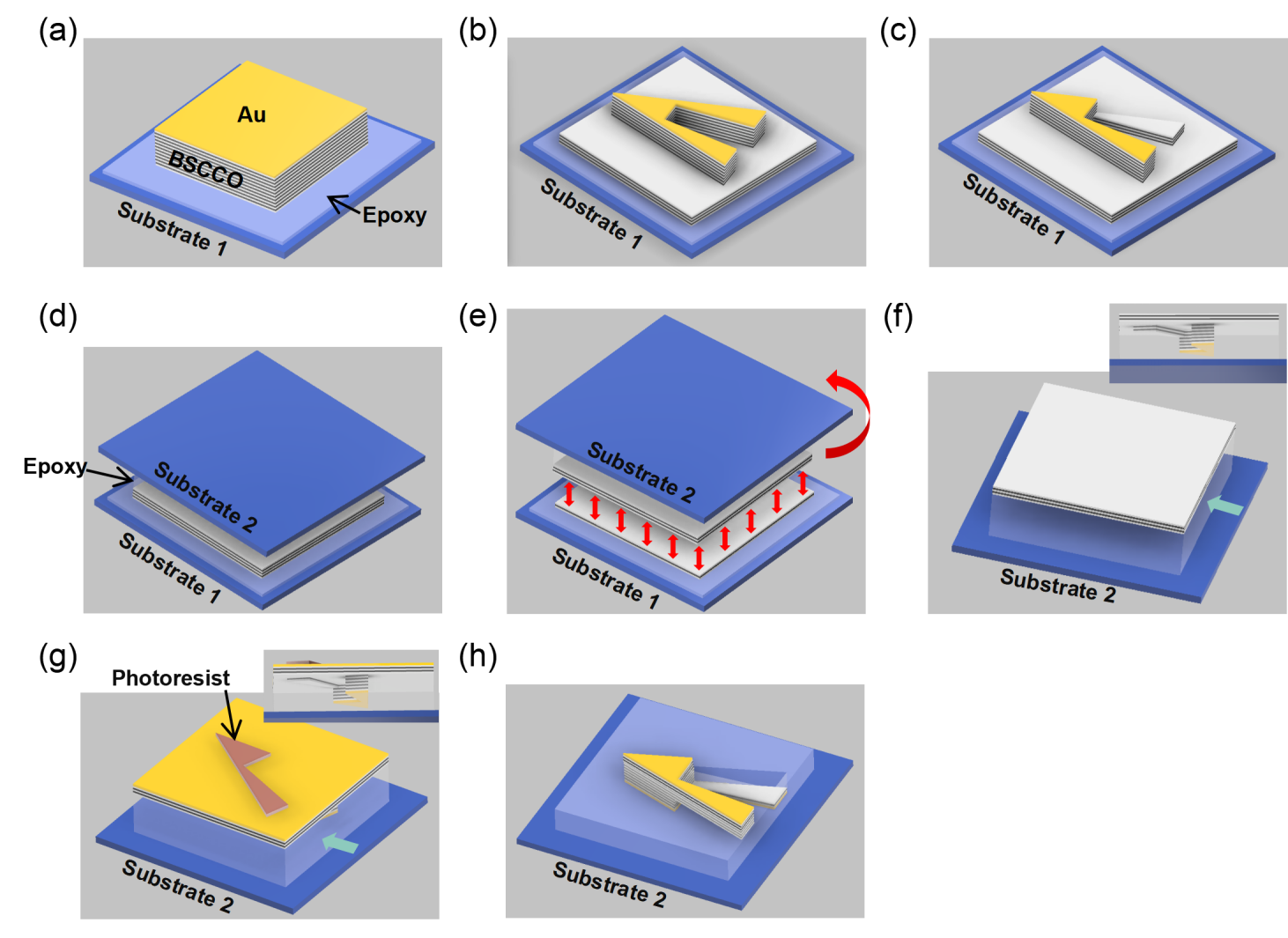


**Supplementary Figure 7.** Fabrication procedure of intrinsic Josephson diodes. (a) Crystal mounting followed by gold film deposition. (b) Patterning of the wedge-shaped crystal mesa. (c) Defining the electrode on one side. (d) Epoxy encapsulation and placement of the 2^nd^ substrate. (e) Splitting two substrates. (f) Flip-transfer of the device onto the 2^nd^ substrate. (g) Deposition of gold film and photolithography to define the final electrode. (h) Ion milling with precise control over the number of stacked intrinsic junctions.


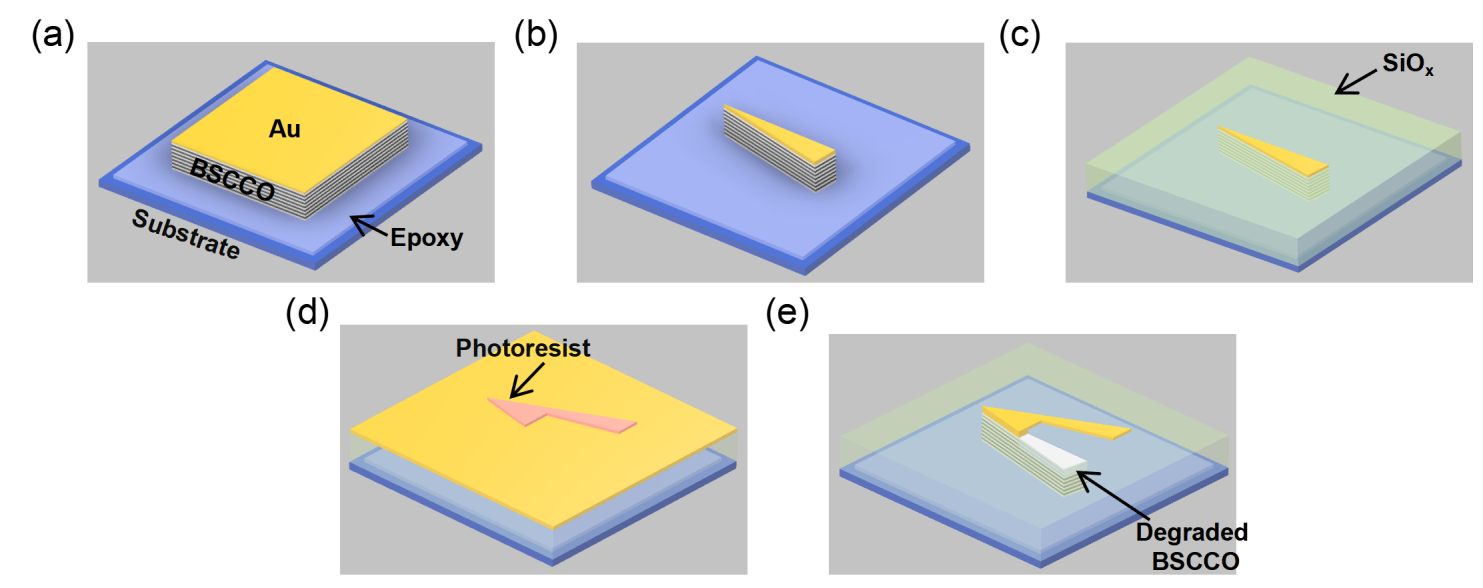


**Supplementary Figure 8.** Fabrication procedure of the surface intrinsic Josephson diodes. (a) Crystal mounting followed by gold film deposition. (b) Patterning of the wedge-shaped stack. (c) Deposition of an insulating layer. (d) Gold film deposition and photolithography. (e) Wet etching of gold film to define electrodes.


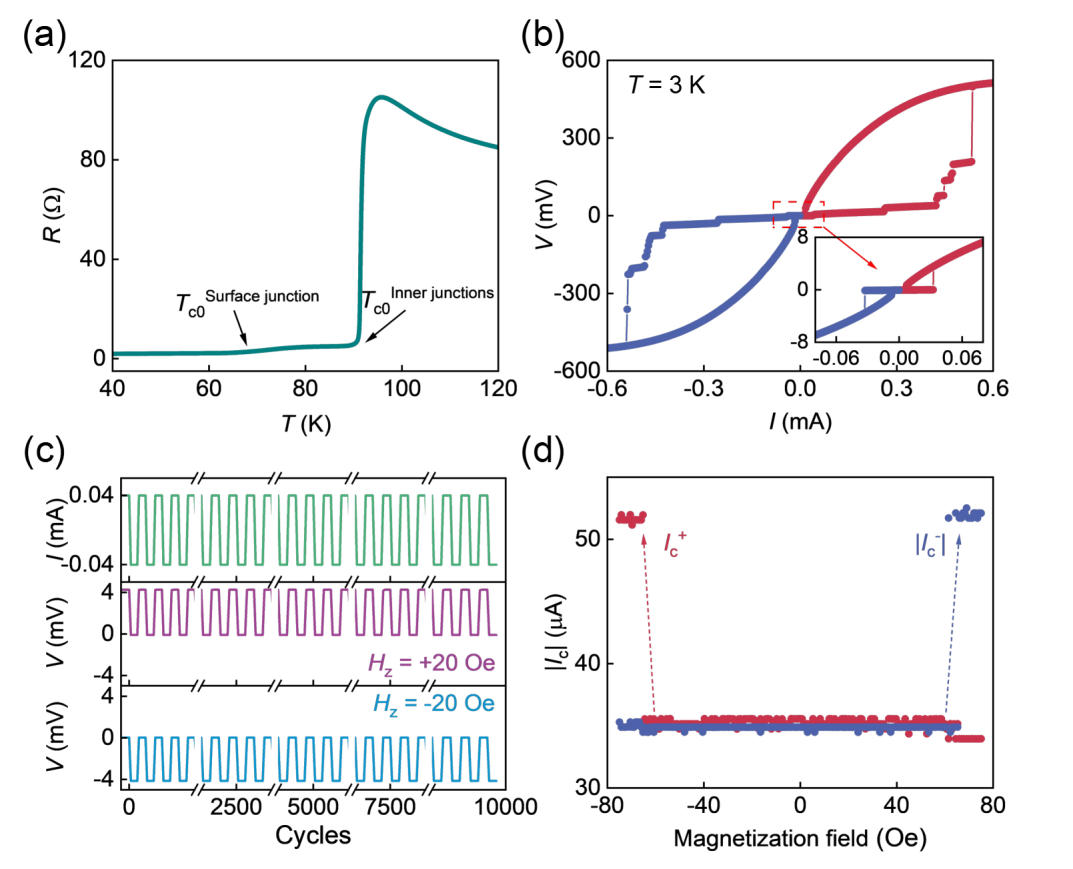


**Supplementary Figure 9.** Transport properties of the surface intrinsic Josephson junction device. (a) Temperature dependence of resistance. The bias current is 10 μA, and voltages were measured by subtracting the signals from positive and negative currents to eliminate offsets. Two distinct resistance kinks correspond to the superconducting transitions of stacked IJJs and the surface IJJ, respectively. (b) Full-range current-voltage characteristics. A voltage jump (~500 mV) indicates the presence of tens of IJJs in the device. The inset shows an enlarged view of the low-bias response, highlighting the characteristic from the surface IJJ. (c) Half-wave rectification at 3 K. Under square-wave current excitation, the device generates stable rectified voltage output under a magnetic of ± 20 Oe. Repeatable rectification is observed for over 10,000 cycles. (d) Magnetization-programmed diode behavior. Critical currents were measured at zero field after applying different magnetization fields. Nonreciprocal zero-field critical currents (*I*_c_^+^: red, |*I*_c_^-^|: blue), emerge when the magnetization field exceeds ±60 Oe.


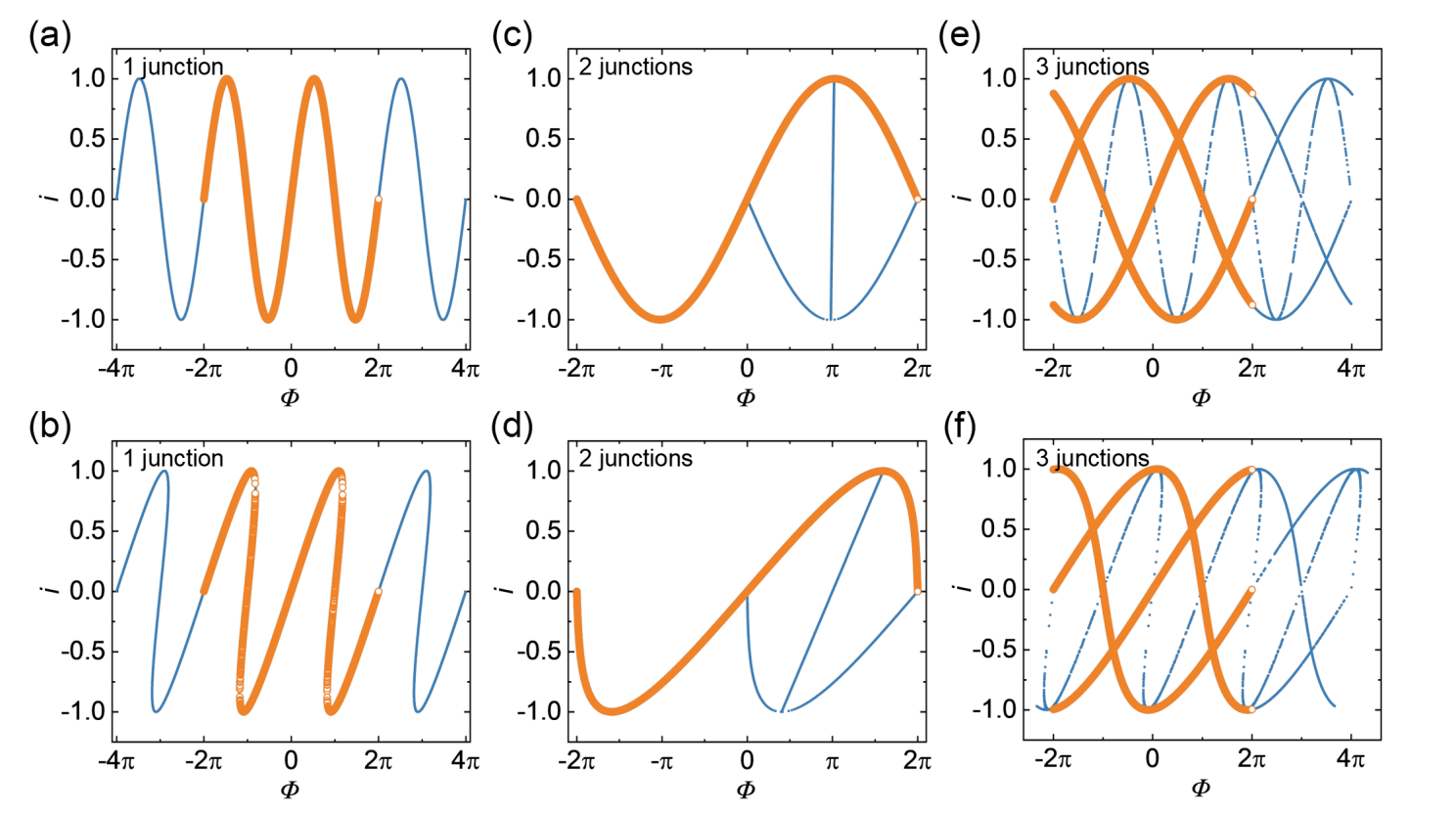


**Supplementary Figure 10.** Comparison between numerical simulation and analytical model. Numerically computed current-phase relations (blue) are compared with analytical results from Eq. (11) (orange) under zero magnetic field. The top and bottom panels correspond to small and large anharmonicity, respectively. Excellent agreement is observed between the two approaches.
